# Integrating pharmacovigilance signals with real-world validation: a study on neurological events associated with PCSK9 inhibitors

**DOI:** 10.3389/fmed.2026.1765715

**Published:** 2026-03-06

**Authors:** Bing Zhu, Qiqi Shao, Jun Cui, Zhenyan Fu, Yitong Ma

**Affiliations:** The First Affiliated Hospital of Xinjiang Medical University, Urumqi, China

**Keywords:** adverse drug events, BCPNN, FAERS, nervous system disorders, PCSK9 inhibitors, ROR

## Abstract

**Background:**

Proprotein convertase subtilisin/kexin type 9 (PCSK9) inhibitors are novel drugs widely used in clinical practice for the treatment of dyslipidemia. However, real-world data regarding their long-term neurological safety and tolerability in large populations remain incomplete. Therefore, we utilized the FAERS and real-world data from Chinese patients to jointly analyze the association between PCSK9 inhibitors and adverse drug events (ADEs) related to nervous system disorders.

**Methods:**

A disproportionality analysis was performed on all ADEs associated with PCSK9 inhibitors in the FAERS database from the third quarter of 2015 to the second quarter of 2025. The reporting odds ratio (ROR) and Bayesian confidence propagation neural network (BCPNN) methods were employed to comprehensively evaluate and screen for statistically significant positive signals of adverse drug reactions related to nervous system disorders. These signals were further validated using follow-up data from 1,203 Chinese coronary heart disease (CHD) patients treated with PCSK9 inhibitors.

**Results:**

A total of 173,622 reports involving at least one PCSK9 inhibitor were identified in FAERS. Statistically significant positive ADEs signals associated with PCSK9 inhibitors included: Memory Impairment [*n* = 1,346, ROR = 1.48 (95% CI 1.4–1.56), IC = 0.55 (IC025 0.47), Bonferroni-*p* = 2.2028e^−42^], Amnesia [*n* = 452, ROR = 1.25 (95% CI 1.14–1.38), IC = 0.32 (IC025 0.18), Bonferroni-*p* = 0.0097], Head Discomfort [*n* = 190, ROR = 1.46 (95% CI 1.26–1.68), IC = 0.54 (IC025 0.32), Bonferroni-*p* = 0.0013], Sinus Headache [*n* = 65, ROR = 2.38 (95% CI 1.86–3.04), IC = 1.23 (IC025 0.84), Bonferroni-*p* = 1.0944e^−08^], and Carotid Artery Occlusion [*n* = 56, ROR = 3.08 (95% CI 2.36–4.02), IC = 1.59 (IC025 1.16), Bonferroni-*p* = 3.2089e^−14^]. Analysis of real-world follow-up data from Chinese CHD patients revealed that, compared to CHD patients not using any PCSK9 inhibitors, those treated with PCSK9 inhibitors exhibited significantly higher incidences of Memory Impairment (*p* < 0.0001) and Head Discomfort (*p* = 0.0027).

**Conclusion:**

Our study highlights that it is essential to recognize the potential risks of adverse neurological reactions, particularly Memory Impairment and Head Discomfort. These findings may assist healthcare professionals in providing more precise and individualized treatment plans.

## Introduction

Atherosclerosis-related diseases (ASD) represent a complex pathological condition characterized by the formation of atherosclerotic plaques within the arterial wall, which contain cholesterol deposits largely attributable to elevated levels of low-density lipoprotein cholesterol (LDL-C). Proprotein convertase subtilisin/kexin type 9 (PCSK9) plays a key role in cholesterol homeostasis, primarily by binding to the low-density lipoprotein receptor (LDL-R) and inducing its lysosomal degradation. Therefore, inhibiting PCSK9 increases the expression of LDL-R on the cell surface, thereby promoting the clearance of LDL-C from the blood (1). Indeed, numerous clinical trials have confirmed the efficacy of PCSK9 inhibitors (PCSK9i) in reducing LDL-C (2–4). Moreover, these agents have significantly improved cardiovascular outcomes in several clinical trials (5, 6). Extensive clinical studies have demonstrated the efficacy of PCSK9 inhibitors in lowering LDL-C, showing significant cardiovascular benefits (7, 8). The FOURIER trial (9) found that the PCSK9 inhibitor evolocumab reduced the incidence of the primary composite endpoint by 1.5% (9.8% vs. 11.3%). The ODYSSEY OUTCOMES trial (10) showed that alirocumab reduced the incidence of the primary composite endpoint by 1.6% (9.5% vs. 11.1%) over 2.8 years in patients with acute coronary syndrome.

Inclisiran, a novel small interfering RNA (siRNA) molecule that inhibits PCSK9 synthesis, provides robust and long-term LDL-C reduction, accompanied by low interindividual variability in the LDL-C-lowering response (11, 12). However, with the expanding clinical use and cumulative duration of PCSK9 inhibitor exposure, concerns regarding their long-term safety, particularly potential neurological effects, have grown. Although early large-scale randomized controlled trials (RCTs) did not centrally report such risks, RCTs are limited by their specific study populations, relatively constrained sample sizes, and follow-up durations, which may not fully capture long-term, rare, or specific types of adverse events in real-world settings. This study innovatively integrates the US FDA Adverse Event Reporting System (FAERS)—a large-scale pharmacovigilance database—with real-world follow-up data from a single-center Chinese cohort of coronary heart disease patients. For the first time, we systematically investigate the potential association between PCSK9 inhibitors and nervous system adverse drug events through a combined approach of disproportionality analysis and empirical validation.

## Methods

### Data source and extraction

The FAERS database serves as a key source for post-marketing safety surveillance of all approved drugs in the United States. It employs the Medical Dictionary for Regulatory Activities (MedDRA) Preferred Term (PT) to define ADEs.^1^ This study extracted all ADEs associated with PCSK9 inhibitors from the FAERS database spanning the third quarter of 2015 to the second quarter of 2025. The original dataset was obtained from the official FDA website.^2^ General information on ADEs related to PCSK9 inhibitors was collected, including age, sex, reporting year, reporting region, reporter type, and outcome. To address the common issue of duplicate case entries in spontaneous reporting systems, this study followed FDA recommendations by retaining only the latest case ID for each unique report. Furthermore, to enhance the accuracy and specificity of signal detection, we applied multiple identical identifiers—including age, sex, occurrence country, event date, drug name, start date, end date, and patient details—to more precisely identify and manually remove duplicate cases among the ADRs that generated positive signals.

The Chinese cohort of coronary heart disease patients is a single-center, retrospective cohort study, with neurological outcomes assessed via telephone follow-up. Data were obtained from the Heart Center of the First Affiliated Hospital of Xinjiang Medical University (February 2022 to May 2025). A total of 1,203 patients were included, comprising 601 CHD patients who were first-time users of PCSK9 inhibitors and 602 CHD patients who did not receive any PCSK9 inhibitors. Inclusion criteria were: (1) age ≥ 18 years; (2) confirmed diagnosis of CHD; and (3) hemodynamic stability. Exclusion criteria included: (1) loss to follow-up; (2) incomplete medical records; (3) history of neurotoxic drug use; and (4) severe anemia, uncontrolled serious infection, or malignancy. Patient demographic and clinical data—including basic information, mobile number, age, sex, history of hypertension, smoking, alcohol use, and laboratory test results—were collected from the hospital’s cardiovascular disease-specific database. In October 2025, telephone follow-up was conducted for the 601 first-time PCSK9 inhibitor users and the 602 non-users to assess the occurrence of statistically significant nervous system adverse drug events identified from the FAERS database, including Memory Impairment, Amnesia, Head Discomfort, Sinus Headache, and Carotid Artery Occlusion. The telephone follow-up was performed by specially trained research staff. All reported positive events were verified by reviewing available outpatient or inpatient medical records within our hospital system. An event was only confirmed if documented in the medical records or if the patient’s description strongly aligned with the Preferred Term definition and its timing post-drug initiation. Before formal data analysis commenced, all identifiable information was deleted and replaced with a unique study code. The study was approved by the Ethics Committee of the First Affiliated Hospital of Xinjiang Medical University (Urumqi, China; Ethical Application Ref: 240104-01), and all participants provided written informed consent.

### Data analysis and signal filtering

A disproportionality analysis was performed to assess the association between nervous system ADEs and PCSK9 inhibitors using the Reporting Odds Ratio (ROR) and Information Component (IC). The IC is derived from Bayesian Confidence Propagation Neural Network (BCPNN) theory, which compares observed versus expected frequencies of drug-ADE associations and incorporates known probability differences in background data to develop sensitive indicators for identifying new disproportionality signals. The ROR is a disproportionality measure that compares the odds of a specific drug-ADE combination being reported versus all other drug-ADE combinations in the database. Given the high specificity of IC and the high sensitivity of ROR in small samples, this study employed both statistical measures to minimize the impact of spurious high ROR values and enable more robust safety signal detection. When using the full database as the control group, an ADE was considered a positive signal only if both the ROR and IC algorithms detected a disproportionality signal. A statistically significant association was defined when the following criteria were met simultaneously: ROR (lower limit of 95% CI > 1, with at least 50 cases), IC025 > 0, and Bonferroni-adjusted *p* < 0.05. Detailed formulas and thresholds for the ROR and IC algorithms are provided in Supplementary Tables S1, S2. In the drug-specific signal analysis (Table 1), to account for the multiplicity of testing inherent in screening for each drug, a stringent Bonferroni correction was applied. The correction factor for each drug (Evolocumab, Alirocumab, Inclisiran) was defined as the total number of unique Preferred Terms (PTs) reported for the respective drug in the FAERS dataset (*n* = 4,331, 2,554, and 1,534, respectively). The reported Bonferroni-corrected *p*-values in Table 1 reflect this adjustment.

**Table 1:** Performance of the five positive signal PTs for individual PCSK9 inhibitors.

Drug/PT	*n*	ROR (95% Cl)	IC (IC025)	*p*	Bonferroni-*p*
Evolocumab					
Memory impairment	1,041	1.39 (1.31–1.48)	0.47 (0.38)	^c^4.4757e^−26^	**1.9384e** ^**−22**^
Amnesia	331	1.12 (1.00–1.24)	0.16 (0.00)	^c^0.0489	>0.9999
Head discomfort	141	1.32 (1.12–1.55)	0.39 (0.15)	^c^0.0013	>0.9999
Sinus headache	45	1.99 (1.49–2.68)	0.98 (0.52)	^c^4.6974e^−06^	**0.0203**
Carotid artery occlusion	43	2.87 (2.12–3.88)	1.50 (1.00)	^c^2.2168e^−12^	**9.6010e** ^**−09**^
Alirocumab					
Memory impairment	237	1.84 (1.62–2.09)	0.87 (0.68)	^c^3.5067e^−21^	**8.9561e** ^**−18**^
Amnesia	108	2.12 (1.76–2.57)	1.08 (0.79)	^c^2.3390e^−15^	**5.9738e** ^**−12**^
Head discomfort	38	2.06 (1.50–2.84)	1.04 (0.54)	^c^9.1179e^−06^	**0.0233**
Sinus headache	19	4.89 (3.11–7.67)	2.28 (1.38)	^f^3.5016e^−08^	**8.9431e** ^**−05**^
Carotid artery occlusion	13	5.00 (2.90–8.62)	2.31 (1.18)	^f^3.7616e^−06^	0.0096
Inclisiran					
Memory impairment	68	2.13 (1.68–2.71)	1.09 (0.72)	^c^3.2025e^−10^	**4.9126e** ^**−07**^
Amnesia	13	1.10 (0.64–1.89)	0.13 (−0.65)	^c^0.8484	>0.9999
Head discomfort	11	2.34 (1.29–4.22)	1.22 (0.24)	^f^0.0092	>0.9999
Sinus headache	1	1.18 (0.17–8.35)	0.23 (−1.93)	^f^0.5731	>0.9999
Carotid artery occlusion	0	—	—	—	—

^c^Chi-Square, ^f^Fisher’s exact test, The Bonferroni correction factor for each drug was the total count of PTs: Evolocumab (4,331), Alirocumab (2,554), Inclisiran (1,534), Bonferroni-*p* < 0.0500 were considered statistically significant and are bolded in the table.

**Figure 1:**
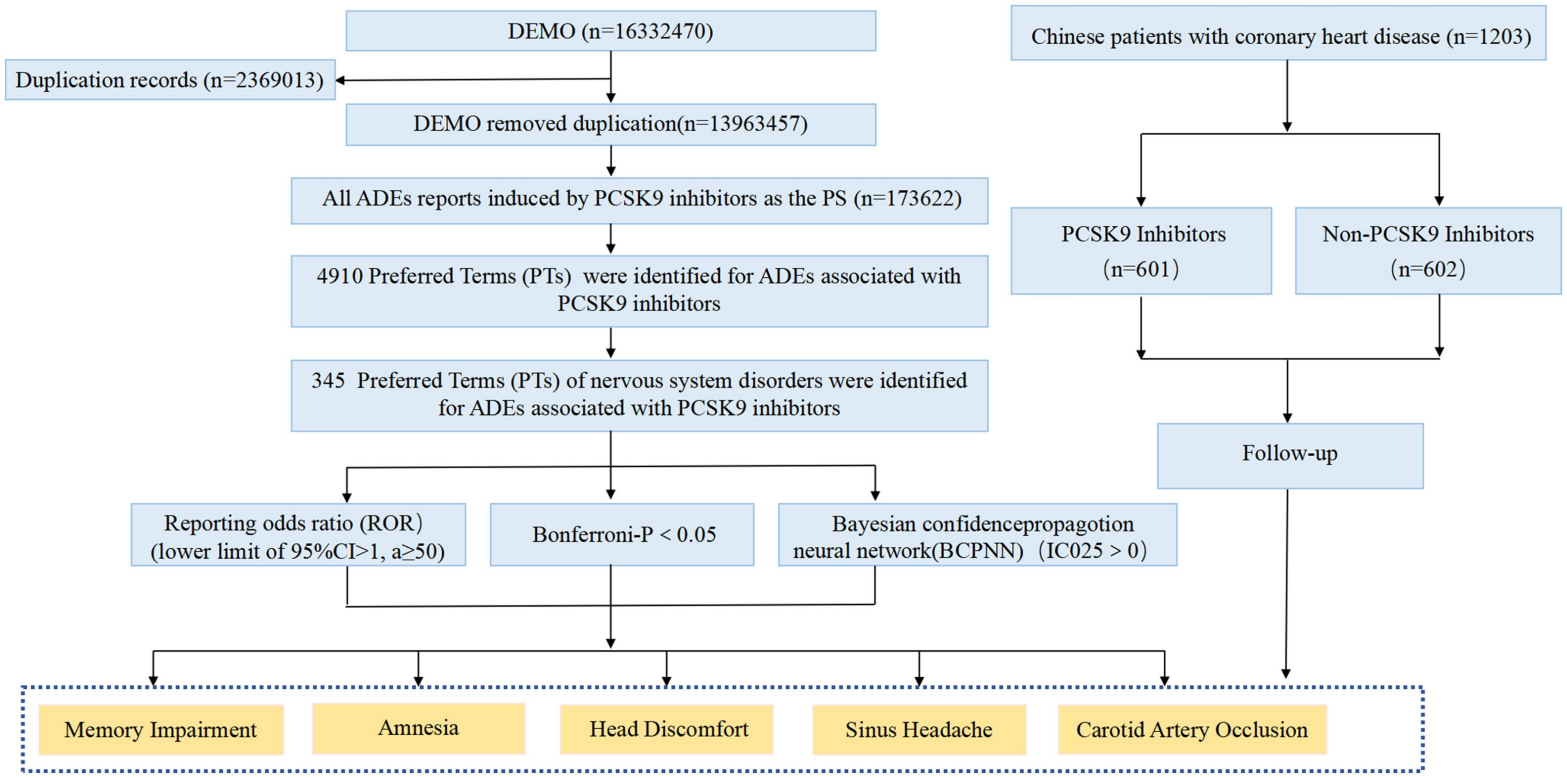
The study process of nervous system disorders associated with PCSK9 inhibitors.

For the Chinese CHD patient data, continuous variables following a normal distribution are expressed as mean ± standard deviation, while non-normally distributed continuous variables are summarized as median (interquartile range). Categorical variables are presented as frequencies and percentages. Independent samples *t*-tests and chi-squared tests were used to assess differences in means and proportions between the two groups. To account for potential confounding and treatment channeling bias, the associations between PCSK9 inhibitor use and each of the five neurological PTs were primarily assessed using multivariable logistic regression models. The models were adjusted for the following pre-specified covariates: age, sex, history of hypertension, smoking status, alcohol use, baseline LDL-C level, and baseline neutrophil count. Results are presented as adjusted odds ratios with 95% confidence intervals. Unadjusted comparisons using chi-square or Fisher’s exact tests are also provided for reference. All analyses and figures were performed using R software version 4.5.0 and SPSS Statistics 25. The study flowchart is illustrated in Figure 1.

## Result

From the third quarter of 2015 to the second quarter of 2025, a total of 173,622 adverse drug event (ADE) reports associated with PCSK9 inhibitors were recorded, including those for Evolocumab (*N* = 146,498), Alirocumab (*N* = 21,565), and Inclisiran (*N* = 5,559). As shown in Table 2, with the increasing widespread use and cumulative exposure time of PCSK9 inhibitors, the number of reported ADEs related to these agents increased annually from 2015 to 2018. Furthermore, the annual number of reported ADEs has remained consistently above 10,000 cases since 2018, underscoring the importance of enhanced attention to the adverse events associated with PCSK9 inhibitors.

**Table 2:** Demographic and clinical characteristics of patients treated with PCSK9 inhibitors in the FAERS database.

Characteristics	PCSK9 inhibitors (total, *n* = 173,622)	Evolocumab (*n* = 146,498)	Alirocumab (*n* = 21,565)	Inclisiran (*n* = 5,559)
Gender, *n* (%)				
Female	92,955 (53.5%)	79,266 (54.1%)	11,043 (51.2%)	2,646 (47.6%)
Male	66,337 (38.2%)	56,888 (38.8%)	7,543 (35.0%)	1906 (34.3%)
Unknown	14,330 (8.3%)	10,344 (7.1%)	2,979 (13.8%)	1,007 (18.1%)
Weight (kg), *n* (%)				
<50	262 (0.2%)	199 (0.1%)	48 (0.2%)	15 (0.3%)
50–100	7,303 (4.2%)	5,519 (3.8%)	1,260 (5.8%)	524 (9.4%)
>100	1,683 (1.0%)	1,319 (0.9%)	261 (1.2%)	103 (1.9%)
Unknown	164,374 (94.7%)	139,461 (95.2%)	19,996 (92.7%)	4,917 (88.5%)
Age (years), *n* (%)				
<18	112 (0.1%)	101 (0.1%)	7 (0.0%)	4 (0.1%)
18–64.9	41,139 (23.7%)	36,043 (24.6%)	4,384 (20.3%)	712 (12.8%)
65–85	72,828 (41.9%)	63,717 (43.5%)	7,434 (34.5%)	1,677 (30.2%)
>85	2,853 (1.6%)	2,592 (1.8%)	211 (1.0%)	50 (0.9%)
Unknown	56,690 (32.7%)	44,045 (30.1%)	9,529 (44.2%)	3,116 (56.1%)
Outcome, *n* (%)				
Congenital anomaly	12 (0.0%)	10 (0.0%)	1 (0.0%)	1 (0.0%)
Death	2,154 (1.2%)	1,602 (1.1%)	406 (1.9%)	146 (2.6%)
Disability	716 (0.4%)	495 (0.3%)	150 (0.7%)	71 (1.3%)
Hospitalization	8,232 (4.7%)	6,009 (4.1%)	1884 (8.7%)	339 (6.1%)
Life-threatening	494 (0.3%)	369 (0.3%)	73 (0.3%)	52 (0.9%)
Other	162,014 (93.3%)	138,013 (94.2%)	19,051 (88.3%)	4,950 (89.0%)
Reporter type, *n* (%)				
Consumer	90,945 (52.4%)	73,449 (50.1%)	14,798 (68.6%)	2,698 (48.5%)
Health professional	10,210 (5.9%)	7,822 (5.3%)	1,064 (4.9%)	1,324 (23.8%)
Pharmacist	9,152 (5.3%)	7,581 (5.2%)	1,403 (6.5%)	168 (3.0%)
Physician	52,614 (30.3%)	48,829 (33.3%)	2,424 (11.2%)	1,361 (24.5%)
Unknown	10,701 (6.2%)	8,817 (6.0%)	1876 (8.7%)	8 (0.1%)
Reported countries, *n* (%)				
US	166,083 (95.7%)	141,673 (96.7%)	19,891 (92.2%)	4,519 (81.3%)
Non-US	7,539(4.3%)	4,825 (3.3%)	1,674 (7.8%)	1,040 (18.7%)
Reporting year, *n* (%)				
2015	886 (0.5%)	663 (0.5%)	223 (1.0%)	0 (0.0%)
2016	6,710 (3.9%)	4,335 (3.0%)	2,375 (11.0%)	0 (0.0%)
2017	8,063 (4.6%)	5,261 (3.6%)	2,802 (13.0%)	0 (0.0%)
2018	48,161 (27.7%)	44,547 (30.4%)	3,614 (16.8%)	0 (0.0%)
2019	13,988 (8.1%)	9,058 (6.2%)	4,930 (22.9%)	0 (0.0%)
2020	10,509 (6.1%)	8,565 (5.8%)	1944 (9.0%)	0 (0.0%)
2021	11,522 (6.6%)	9,895 (6.8%)	1,626 (7.5%)	1 (0.0%)
2022	13,416 (7.7%)	11,329 (7.7%)	1,392 (6.5%)	695 (12.5%)
2023	26,167 (15.1%)	22,823 (15.6%)	1,306 (6.1%)	2038 (36.7%)
2024	29,375 (16.9%)	26,387 (18.0%)	1,122 (5.2%)	1866 (33.6%)
2025	4,825 (2.8%)	3,635 (2.5%)	231 (1.1%)	959 (17.3%)

The proportion of ADEs for all three PCSK9 inhibitors was higher in females than in males (PCSK9 inhibitors overall: 53.5% vs. 38.2%; Evolocumab: 54.1% vs. 38.8%; Alirocumab: 51.2% vs. 35.0%; Inclisiran: 47.6% vs. 34.3%). The age group most affected by adverse events was 65–85 years across all agents (PCSK9 inhibitors overall: 41.9%; Evolocumab: 43.5%; Alirocumab: 34.5%; Inclisiran: 30.2%). The majority of reports originated from the United States (PCSK9 inhibitors overall: 95.7%; Evolocumab: 96.7%; Alirocumab: 92.2%; Inclisiran: 81.3%), and were primarily submitted by consumers (PCSK9 inhibitors overall: 52.4%; Evolocumab: 50.1%; Alirocumab: 58.6%; Inclisiran: 48.5%).

For PCSK9 inhibitors, there were 42 Preferred Terms (PTs) of adverse events in the Nervous System Disorders organ category reported in no fewer than 50 cases (Table 3). Among these, the most frequently reported adverse events were Headache (*n* = 3,481), followed by Dizziness (*n* = 2,748) and Memory Impairment (*n* = 1,346). Using two algorithms, ROR and BCPNN, to detect positive signals among the 42 PTs in Nervous System Disorders, seven PTs were identified as positive signals by both methods. These included Memory Impairment [*n* = 1,346, ROR = 1.48 (95% CI 1.4–1.56), IC = 0.55 (IC025 0.47)], Amnesia [*n* = 452, ROR = 1.25 (95% CI 1.14–1.38), IC = 0.32 (IC025 0.18)], Head Discomfort [*n* = 190, ROR = 1.46 (95% CI 1.26–1.68), IC = 0.54 (IC025 0.32)], Transient Ischaemic Attack [*n* = 187, ROR = 1.18 (95% CI 1.02–1.36), IC = 0.23 (IC025 0.02)], Sciatica [*n* = 133, ROR = 1.25 (95% CI 1.05–1.48), IC = 0.32 (IC025 0.07)], Sinus Headache [*n* = 65, ROR = 2.38 (95% CI 1.86–3.04), IC = 1.23 (IC025 0.84)], and Carotid Artery Occlusion [*n* = 56, ROR = 3.08 (95% CI 2.36–4.02), IC = 1.59 (IC025 1.16)].

**Table 3:** PTs related to nervous system ADEs for PCSK9 inhibitors (*n* ≥ 50).

PT	*n*	ROR (95% Cl)	IC (IC025)
Headache	3,481	0.87 (0.84–0.9)	−0.20 (−0.25)
Dizziness	2,748	0.90 (0.86–0.93)	−0.15 (−0.21)
Memory impairment	1,346	1.48 (1.40–1.56)	0.55 (0.47)
Cerebrovascular accident	839	1.03 (0.97–1.11)	0.05 (−0.05)
Hypoaesthesia	738	0.83 (0.77–0.89)	−0.27 (−0.37)
Paraesthesia	720	0.77 (0.71–0.82)	−0.38 (−0.49)
Tremor	478	0.50 (0.45–0.54)	−1.00 (−1.13)
Burning sensation	457	1.10 (1.00–1.20)	0.13 (0.00)
Amnesia	452	1.25 (1.14–1.38)	0.32 (0.18)
Neuropathy peripheral	421	0.65 (0.59–0.72)	−0.62 (−0.76)
Lethargy	362	1.09 (0.98–1.21)	0.12 (−0.03)
Somnolence	357	0.29 (0.26–0.32)	−1.79 (−1.94)
Balance disorder	332	0.63 (0.56–0.70)	−0.67 (−0.83)
Migraine	304	0.48 (0.43–0.54)	−1.05 (−1.21)
Dysgeusia	264	0.61 (0.54–0.68)	−0.72 (−0.89)
Loss of consciousness	251	0.35 (0.31–0.40)	−1.49 (−1.67)
Cognitive disorder	232	0.78 (0.69–0.89)	−0.35 (−0.54)
Syncope	209	0.36 (0.31–0.41)	−1.47 (−1.66)
Head discomfort	190	1.46 (1.26–1.68)	0.54 (0.32)
Transient Ischaemic attack	187	1.18 (1.02–1.36)	0.23 (0.02)
Dysstasia	182	0.99 (0.86–1.15)	−0.01 (−0.23)
Dementia	178	0.96 (0.82–1.11)	−0.06 (−0.28)
Disturbance in attention	167	0.51 (0.44–0.60)	−0.96 (−1.18)
Neuralgia	141	0.86 (0.73–1.02)	−0.21 (−0.45)
Speech disorder	140	0.46 (0.39–0.54)	−1.11 (−1.35)
Sciatica	133	1.25 (1.05–1.48)	0.32 (0.07)
Presyncope	112	0.74 (0.62–0.89)	−0.43 (−0.69)
Hypersomnia	106	0.58 (0.48–0.71)	−0.77 (−1.04)
Brain fog	106	1.12 (0.92–1.35)	0.16 (−0.12)
Seizure	101	0.10 (0.09–0.13)	−3.24 (−3.51)
Restless legs syndrome	83	0.76 (0.61–0.94)	−0.40 (−0.71)
Aphasia	81	0.46 (0.37–0.57)	−1.11 (−1.42)
Mental impairment	81	0.56 (0.45–0.69)	−0.83 (−1.15)
Nervous system disorder	71	0.60(0.48–0.76)	−0.72 (−1.05)
Sinus headache	65	2.38 (1.86–3.04)	1.23 (0.84)
Taste disorder	61	0.36 (0.28–0.47)	−1.45 (−1.81)
Nerve compression	61	1.02 (0.79–1.31)	0.03 (−0.34)
Movement disorder	59	0.34 (0.26–0.44)	−1.55 (−1.91)
Cerebral infarction	56	0.40 (0.31–0.52)	−1.31 (−1.67)
Carotid artery occlusion	56	3.08 (2.36–4.02)	1.59 (1.16)
Dysarthria	55	0.27 (0.20–0.35)	−1.9 (−2.27)
Cerebral haemorrhage	51	0.24 (0.18–0.31)	−2.08 (−2.46)

**Figure 2:**
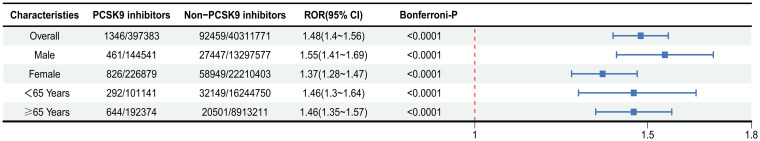
Subgroup analyses of memory impairment by gender and age.

**Figure 3:**
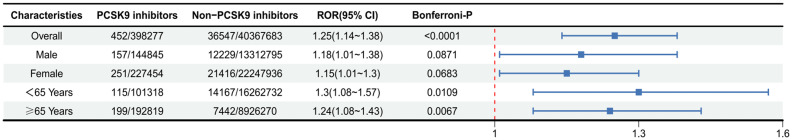
Subgroup analyses of amnesia by gender and age.

**Figure 4:**
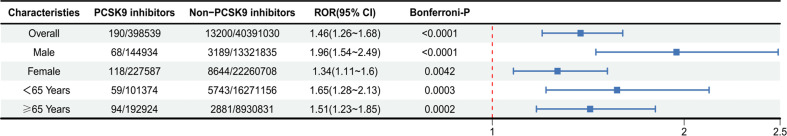
Subgroup analyses of head discomfort by gender and age.

**Figure 5:**
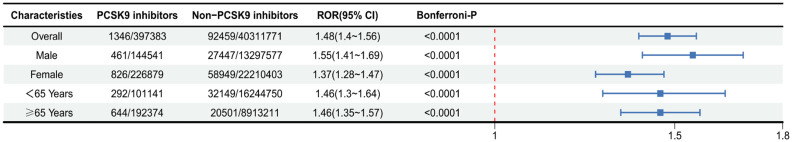
Subgroup analyses of sinus headache by gender and age.

**Figure 6:**
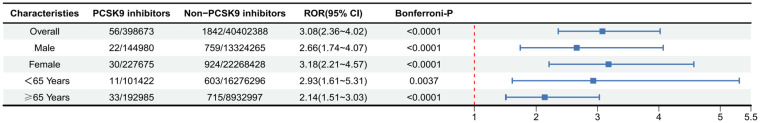
Subgroup analyses of carotid artery occlusion by gender and age.

After applying the chi-square test and Bonferroni correction [The Bonferroni correction factor for PCSK9 Inhibitors were the total count of PTs (4,910)], only five of these seven PTs demonstrated statistical significance: Memory Impairment (Bonferroni-*p* = 2.2028e^−42^), Amnesia (Bonferroni-*p* = 0.0097), Head Discomfort (Bonferroni-*p* = 0.0013), Sinus Headache (Bonferroni-*p* = 1.0944e^−08^), and Carotid Artery Occlusion (Bonferroni-*p* = 3.2089e^−14^) (Table 4). In the baseline table of positive PTs, it was observed that these five significant signals predominantly occurred in individuals aged over 65 years: Memory Impairment (47.8%), Amnesia (44%), Head Discomfort (49.4%), Sinus Headache (47.7%), and Carotid Artery Occlusion (58.9%). Moreover, these signals were notably more frequent in females than in males: Memory Impairment (61.4% vs. 34.2%), Amnesia (55.5% vs. 34.7%), Head Discomfort (62.1% vs. 35.8%), Sinus Headache (61.3% vs. 30.8%), and Carotid Artery Occlusion (53.6% vs. 39.3%) (see Supplementary Table S3). Subgroup analyses by age and sex were subsequently conducted for each positive signal. The results indicated that Memory Impairment, Head Discomfort, Sinus Headache, and Carotid Artery Occlusion were positive signals in both age and sex subgroups, all of which were statistically significant (Figures 2, 4–6). For Amnesia, it was identified as a positive signal in the sex subgroup but did not reach statistical significance; however, it remained a positive signal in the age subgroup with statistical significance (Figure 3).

**Table 4:** PTs for PCSK9 inhibitors meeting positive signal criteria by both ROR and BCPNN algorithms.

PT	*n*	ROR (95% Cl)	IC (IC025)	*p*	Bonferroni-*p*
Memory impairment	1,346	1.48 (1.4–1.56)	0.55 (0.47)	^c^4.4864e^−46^	**2.2028e** ^**−42**^
Amnesia	452	1.25 (1.14–1.38)	0.32 (0.18)	^c^1.9779e^−06^	**0.0097**
Head discomfort	190	1.46 (1.26–1.68)	0.54 (0.32)	^c^2.5564e^−07^	**0.0013**
Transient Ischaemic attack	187	1.18 (1.02–1.36)	0.23 (0.02)	^c^0.0284	>0.9999
Sciatica	133	1.25 (1.05–1.48)	0.32 (0.07)	^c^0.0115	>0.9999
Sinus headache	65	2.38 (1.86–3.04)	1.23 (0.84)	^c^2.2290e^−12^	**1.0944e** ^**−08**^
Carotid artery occlusion	56	3.08 (2.36–4.02)	1.59 (1.16)	^c^6.5355e^−18^	**3.2089e** ^**−14**^

^c^Chi-Square, The Bonferroni correction factor for PCSK9 Inhibitors were the total count of PTs (4,910), Bonferroni-*p* < 0.0500 were considered statistically significant and are bolded in the table.

Analysis of five statistically significant positive signal Preferred Terms (PTs) across three drugs—Evolocumab, Alirocumab, and Inclisiran—revealed that Memory Impairment was a statistically significant positive signal in Evolocumab [*n* = 1,041, ROR = 1.39 (95% CI 1.31–1.48), IC = 0.47 (IC025 0.38), Bonferroni-*p* = 1.9384e^−22^], Alirocumab [*n* = 237, ROR = 1.84 (95% CI 1.62–2.09), IC = 0.87 (IC025 0.68), Bonferroni-*p* = 8.9561e^−18^], and Inclisiran [*n* = 68, ROR = 2.13 (95% CI 1.68–2.71), IC = 1.09 (IC025 0.72), Bonferroni-*p* = 4.9126e^−07^]. Amnesia was a statistically significant positive signal only in Alirocumab [*n* = 108, ROR = 2.12 (95% CI 1.76–2.57), IC = 1.08 (IC025 0.79), Bonferroni-*p* = 5.9738e^−12^], but not in Evolocumab or Inclisiran, where it showed no significant signal. Head Discomfort was a statistically significant positive signal in Alirocumab [*n* = 38, ROR = 2.06 (95% CI 1.50–2.84), IC = 1.04 (IC025 0.54), Bonferroni-*p* = 0.0233]. Sinus Headache was a statistically significant positive signal in Evolocumab [*n* = 45, ROR = 1.99 (95% CI 1.49–2.68), IC = 0.98 (IC025 0.52), Bonferroni-*p* = 0.0203] and Alirocumab [*n* = 19, ROR = 4.89 (95% CI 3.11–7.67), IC = 2.28 (IC025 1.38), Bonferroni-*p* = 8.9431e^−05^], but not in Inclisiran, where no significant signal was observed. Carotid Artery Occlusion was a statistically significant positive signal in Evolocumab [*n* = 43, ROR = 2.87 (95% CI 2.12–3.88), IC = 1.50 (IC025 1.00), Bonferroni-*p* = 9.6010e^−09^] and Alirocumab [*n* = 13, ROR = 5.00 (95% CI 2.90–8.62), IC = 2.31 (IC025 1.18), Bonferroni-*p* = 0.0096], but was not reported for Inclisiran (Table 1).

In a follow-up study of adverse drug events related to nervous system disorders in 601 Chinese coronary heart disease patients using PCSK9 inhibitors and 602 not using any PCSK9 inhibitors, Memory Impairment (*p* < 0.0001) and Head Discomfort (*p* = 0.0027) showed statistically significant differences, while Amnesia (*p* = 0.1079), Sinus Headache (*p* = 0.0795), and Carotid Artery Occlusion (*p* = 0.2176) did not reach statistical significance. After adjusting for age, sex, hypertension, smoking, alcohol use, baseline LDL-C, and neutrophil count, the use of PCSK9 inhibitors remained independently associated with a significantly higher risk of Memory Impairment (Adjusted-OR = 4.729, 95% CI 3.010–7.428, *p* < 0.001) and Head Discomfort (Adjusted-OR = 1.984, 95% CI 1.158–3.399, *p* = 0.013) (Table 5).

**Table 5:** Adverse drug events for the five positive signal PTs in Chinese coronary heart disease patients using PCSK9 inhibitors.

Parameters	PCSK9 inhibitors (*n* = 601)	Non-PCSK9 inhibitors (*n* = 602)	*p*	Adjusted-*p*	Adjusted-OR (95% CI)
Memory impairment (*n*, %)			**<0.001** ^**c**^	**<0.001**	**4.729 (3.010–7.428)**
Yes	106 (17.64)	26 (4.32)			
No	495 (82.36)	576 (95.68)			
Amnesia (*n*, %)			0.108^f^	0.113	3.636 (0.737–17.948)
Yes	7 (1.16)	2 (0.33)			
No	594 (98.84)	600 (99.67)			
Head discomfort (*n*, %)			**0.003** ^**c**^	**0.013**	**1.984 (1.158–3.399)**
Yes	46 (7.65)	22 (3.65)			
No	555 (92.35)	580 (96.35)			
Headache			0.080^c^	0.134	1.659 (0.856–3.217)
Yes	26 (4.33)	15 (2.49)			
No	575 (95.67)	587 (97.51)			
Carotid artery occlusion (*n*, %)			0.218^f^	0.128	5.666 (0.608–52.840)
Yes	4 (0.67)	1 (0.17)			
No	597 (99.33)	601 (99.83)			

^c^Chi-Square, ^f^Fisher’s exact test, Adjusted-*p* values and Adjusted-ORs are derived from multivariable logistic regression models adjusting for age, sex, hypertension, smoking, alcohol use, baseline LDL-C, and neutrophil count, Adjusted-*p* < 0.050 were considered statistically significant and are bolded in the table.

## Discussion

This study innovatively integrated the US FDA Adverse Event Reporting System (FAERS)—a large-scale pharmacovigilance database—with real-world follow-up data from a single-center Chinese cohort of coronary heart disease patients. For the first time, we systematically investigated the potential association between PCSK9 inhibitors and nervous system adverse drug events using a combined approach of disproportionality analysis and empirical validation. Our analysis not only revealed statistically significant signal associations between PCSK9 inhibitors and specific nervous system adverse events (such as Memory Impairment and Head Discomfort) but also provided detailed characterization across different drug subtypes and demographic subgroups, with partial validation of signals in a Chinese population. This offers important and novel evidence for the clinical safe application of these drugs. Through mining the FAERS database, we identified five statistically significant positive neurological signals associated with PCSK9 inhibitors, including Memory Impairment, Amnesia, Head Discomfort, Sinus Headache, and Carotid Artery Occlusion. Notably, in our real-world Chinese cohort, the signals for Memory Impairment and Head Discomfort were robustly validated even after rigorous adjustment for multiple clinical confounders (Adjusted-OR = 4.729 and 1.984, respectively), significantly enhancing the credibility of their clinical relevance and underscoring their warning significance.

Memory Impairment was one of the most prominent signals in this study. It constituted a substantial number of reports (*n* = 1,346) in the overall PCSK9 inhibitor data and showed stable and significant positive signals across all three drugs: Evolocumab, Alirocumab, and Inclisiran. The Amnesia signal was significant only for Alirocumab but not stable for Evolocumab and Inclisiran, suggesting that differences in molecular structure, pharmacokinetics, tissue distribution, and potential off-target effects among different PCSK9 inhibitors might lead to variations in their adverse event profiles, or that factors such as user population characteristics and reporting biases for different drugs could be involved. Research has found that PCSK9 is involved in various physiological processes in the central nervous system (CNS), such as cholesterol regulation, apoptosis, neurogenesis, neuronal differentiation, and neuroinflammation (13–15). Abnormal cholesterol metabolism in the brain is associated with various neurodegenerative diseases and cognitive dysfunction (16–20). Conventionally, PCSK9 is thought to act primarily on hepatic LDLR via the circulatory system; however, due to the blood–brain barrier (21), its direct central effects have been questioned. Under physiological conditions, plasma lipoproteins and circulating PCSK9 cannot cross the blood–brain barrier (22). Only locally expressed PCSK9 might affect the abundance of LDL receptor family members, such as apolipoprotein E receptor 2 (apoER2) (19, 23) and the very-low-density lipoprotein receptor (VLDLR) (24), thereby influencing the Reelin signaling pathway, which plays a key role in synaptic plasticity, neuronal migration, and memory formation (25, 26). Most lipids in the nervous system are located in the myelin sheath, a specialized membrane that forms a multi-layered sheath around axons exclusively in the central and peripheral nervous systems. Myelin is characterized by a very high lipid/protein ratio and is particularly enriched in cholesterol (40%) (27). The unique lipid composition of myelin allows for the rapid saltatory conduction of nerve impulses and provides nutritional support to axons; even slight alterations can change adhesive properties and lead to structural disruption (28) and severe neurological diseases (29). Therefore, long-term, potent inhibition of PCSK9 might interfere with cholesterol metabolism in the CNS through direct or indirect pathways, subsequently affecting neuronal synapses and myelin sheath function—this could be the potential mechanism behind the increased risk of Memory Impairment and Amnesia. Notably, in the baseline characteristics, patients aged over 65 accounted for 47.8% of Memory Impairment reports, and females accounted for 61.4%, suggesting that the elderly population might be more susceptible due to age-related cognitive decline. Subsequent age and gender subgroup analyses for Memory Impairment revealed that it remained a positive signal with statistical significance across both age and gender subgroups. These subgroup analyses provide a deeper perspective for understanding these safety signals, hinting that this might be a class effect rather than specific to a single PCSK9 inhibitor. Consequently, closer monitoring and follow-up are warranted for elderly and female patients during the clinical use of PCSK9 inhibitors.

Head Discomfort and Sinus Headache represent another noteworthy set of signals. Head Discomfort was validated in both FAERS and the Chinese cohort, while Sinus Headache showed a very strong signal strength in FAERS (ROR = 2.38). Although these symptoms are typically not considered serious adverse events, they directly impact patient medication experience and adherence. Their underlying mechanisms are likely more complex and non-specific. The direct impact of PCSK9 inhibitors on the CNS might be limited, as circulating PCSK9 and its monoclonal antibodies (e.g., evolocumab, alirocumab) are unlikely to efficiently penetrate the blood–brain barrier (BBB) (30–32). Therefore, the observed neurological adverse events are more likely to stem from indirect effects caused by systemic alterations in lipid metabolism. PCSK9 regulates plasma LDL-C levels by degrading LDLR, and lipid homeostasis is crucial for maintaining neurocyte membrane integrity, myelin structure, and energy supply (33–35). Furthermore, in the peripheral nervous system, PCSK9 influences fatty acid uptake and mitochondrial function in Schwann cells by regulating CD36. Its dysregulation might lead to lipid overload, mitochondrial stress, and small fiber dysfunction (36, 37), which could partially explain symptoms like Head Discomfort and Sinus Headache observed in this study.

Carotid Artery Occlusion was one of the stronger signals in this study (ROR = 3.08, IC = 1.59), yet its mechanism remains unclear. The patient population using PCSK9 inhibitors inherently comprises individuals at high or very high risk for atherosclerotic cardiovascular disease, who already face a significant background risk of disease progression. Although statistical analysis attempts to correct for this bias by comparing against the background incidence rate across the entire database, residual confounding is difficult to eliminate entirely. Moreover, this signal could not be validated in the Chinese real-world cohort (*p* = 0.2176). Although PCSK9 inhibitors have been proven to significantly reduce the risk of atherosclerotic cardiovascular events, PCSK9 itself plays complex roles in vascular inflammation, plaque stability, and thrombogenesis (38, 39). Whether this signal reflects the natural disease progression or a drug-specific effect requires further investigation through rigorously designed epidemiological studies.

Separate analyses of different PCSK9 inhibitors revealed potential differences in their safety profiles. Alirocumab demonstrated stronger signal intensities (higher ROR and IC values) for multiple positive signals (e.g., Amnesia, Sinus Headache, Carotid Artery Occlusion) compared to Evolocumab. Whether this difference stems from subtle variations in molecular structure, PCSK9 binding epitopes, immunogenicity, etc., between the monoclonal antibodies (Evolocumab vs. Alirocumab) warrants further exploration. In this study, Inclisiran showed positive signals for Memory Impairment and Head Discomfort. However, due to its later market entry, the number of reports in the database was far fewer than for the other two drugs. Consequently, some rare events (like Carotid Artery Occlusion) had no reports, and some signals (like Sinus Headache) did not reach statistical significance due to an insufficient number of cases. This implies that evaluating the neurological safety of Inclisiran still requires longer time and larger sample sizes.

## Limitations

When interpreting the results of this study, several inherent limitations must be acknowledged: First, FAERS data are subject to under-reporting, missing information, and potential channeling bias. Second, the Chinese cohort was a single-center study, potentially limiting the representativeness of the sample. The sample size was insufficient for detecting rare events, and the observational nature restricts causal inference, although the case–control design and statistical adjustments enhance the robustness of the findings. Third, this study is primarily an observational epidemiological analysis; the exact pathophysiological mechanisms underlying the observed associations still require further elucidation through basic experiments and targeted preclinical research.

## Conclusion

In summary, by comprehensively utilizing an international pharmacovigilance database and Chinese real-world data, this study systematically reveals, for the first time, significant association signals between PCSK9 inhibitors and specific nervous system adverse events, particularly Memory Impairment and Head Discomfort. These signals were more prominent in elderly and female patients and exhibited variations among different PCSK9 inhibitors. While signals for serious events like Carotid Artery Occlusion require cautious interpretation and confirmation through further research, the signals for Memory Impairment and Head Discomfort, validated through multiple dimensions, hold high clinical relevance. This study addresses the gap in evidence regarding the long-term neurological safety of PCSK9 inhibitors, emphasizes the importance of maintaining vigilance and enhancing monitoring in clinical application, and aims to achieve the optimal balance between cardiovascular risk reduction and medication safety for patients, ultimately promoting the precise and individualized use of PCSK9 inhibitors. Future research should focus on elucidating the biological mechanisms and precisely quantifying these risks in broader populations.

## Data Availability

The raw data supporting the conclusions of this article will be made available by the authors, without undue reservation.

## Footnotes

1
https://www.meddra.org/

2
https://fis.fda.gov/extensions/FPD-QDE-FAERS/FPD-QDE-FAERS.html
