# Supplementary material for: Integrating pharmacovigilance signals with real-world validation: a study on neurological events associated with PCSK9 inhibitors
Source: Front Med (Lausanne). 2026 Mar 6;13:1765715. doi: 10.3389/fmed.2026.1765715 (PMC13002627; doi:10.3389/fmed.2026.1765715)
Supplement: Supplementary file 1 [file Table_1.DOCX]

**S1** **Fourfold table of disproportionality measures**

|  | **Target ADEs** | **Other ADEs** |
| --- | --- | --- |
| **Target drugs** | **a** | **b** |
| **Other drugs** | **c** | **d** |

**S2 Formulas and signal detection criterias for ROR, and BCPNN**

|  | **Formula** | **Signal Detection Criterias** |
| --- | --- | --- |
| **ROR** | **ROR =** $\text{(}\text{a}\text{/}\text{c}\text{)/(}\text{b}\text{/}\text{d}\text{) }$**=** $\text{ad}\text{/}\text{bc}$  **95%CI=** $\text{e}^{\text{ln}\left( \text{ROR} \right)\text{ }\text{±}\text{ }\text{1.96*}\sqrt{\text{(}\frac{\text{1}}{\text{a}}\text{ }\text{+}\text{ }\frac{\text{1}}{\text{b}}\text{ }\text{+}\text{ }\frac{\text{1}}{\text{c}}\text{ }\text{+}\text{ }\frac{\text{1}}{\text{d}}\text{)}}}$ | **lower limit of 95%CI>1, a≥50** |
| **BCPNN** | **IC =** $\text{log}_{\text{2}} \text{(}\text{N}_{\text{observed}}\text{+0.5)/(}\text{N}_{\text{expected}}\text{+0.5)}$  **N _expected_ =** $\text{(}\text{N}_{\text{drug}}\text{*}\text{N}_{\text{effect}}\text{)/}\text{N}_{\text{total}}$  **IC025 =** $\text{IC}\text{-}\text{3.3*}\left( \text{N}_{\text{observed}}\text{+0.5} \right)^{\text{-}\text{ }\frac{\text{1}}{\text{2}}}\text{-}\text{2*}\left( \text{N}_{\text{observed}}\text{+0.5} \right)^{\text{-}\text{ }\frac{\text{3}}{\text{2}}}$ | **IC025＞0** |

N _observed_ is the actual number of case reports for the drug–adverse event combination.

N _expected_ is the number of case reports expected for the drug–adverse event combination.

N _drug_ is the number of case reports for the drug, regardless of adverse events.

N _effect_ is the number of case reports for the adverse event, regardless of drug.

N _total_ is the total number of case reports in the database.

**S3 Baseline Characteristics of the Five Positive Signal PTs**

|  | Memory Impairment (N=1346) | Amnesia (N=452) | Head Discomfort (N=190) | Sinus Headache (N=65) | Carotid Artery Occlusion (N=56) |
| --- | --- | --- | --- | --- | --- |
| Gender, n (%) |  |  |  |  |  |
| Female | 826 (61.4%) | 251 (55.5%) | 118 (62.1%) | 40 (61.5%) | 30 (53.6%) |
| Male | 461 (34.2%) | 157 (34.7%) | 68 (35.8%) | 20 (30.8%) | 22 (39.3%) |
| Unknown | 59 (4.4%) | 44 (9.7%) | 4 (2.1%) | 5 (7.7%) | 4 (7.1%) |
| Weight (kg), n (%) |  |  |  |  |  |
| ＜50 | 1 (0.1%) | 1 (0.2%) | 1 (0.5%) | 0 (0%) | 1 (1.8%) |
| 50～100 | 101 (7.5%) | 40 (8.8%) | 24 (12.6%) | 4 (6.2%) | 3 (5.4%) |
| ＞100 | 11 (0.8%) | 9 (2.0%) | 0 (0%) | 2 (3.1%) | 2 (3.6%) |
| Unknown | 1233 (91.6%) | 402 (88.9%) | 165 (86.8%) | 59 (90.8%) | 50 (89.3%) |
| Age (years), n (%) |  |  |  |  |  |
| <18 | 0 (0%) | 0 (0%) | 0 (0%) | 0 (0%) | 0 (0%) |
| 18～64.9 | 292 (21.7%) | 115 (25.4%) | 59 (31.1%) | 20 (30.8%) | 11 (19.6%) |
| 65～85 | 605 (44.9%) | 192 (42.5%) | 89 (46.8%) | 30 (46.2%) | 32 (57.1%) |
| >85 | 39 (2.9%) | 7 (1.5%) | 5 (2.6%) | 1 (1.5%) | 1 (1.8%) |
| Unknown | 410 (30.5%) | 138 (30.5%) | 37 (19.5%) | 14 (21.5%) | 12 (21.4%) |
| Outcome, n (%) |  |  |  |  |  |
| Congenital Anomaly | 2 (0.1%) | 0 (0%) | 0 (0%) | 0 (0%) | 0 (0%) |
| Death | 2 (0.1%) | 1 (0.2%) | 0 (0%) | 0 (0%) | 0 (0%) |
| Disability | 16 (1.2%) | 5 (1.1%) | 2 (1.1%) | 1 (1.5%) | 0 (0%) |
| Hospitalization | 107 (7.9%) | 32 (7.1%) | 9 (4.7%) | 3 (4.6%) | 17 (30.4%) |
| Life-Threatening | 4 (0.3%) | 4 (0.9%) | 1 (0.5%) | 0 (0%) | 1 (1.8%) |
| Other | 1215 (90.3%) | 410 (90.7%) | 178 (93.7%) | 61 (93.8%) | 38 (67.9%) |
| Reporter Type, n (%) |  |  |  |  |  |
| Consumer | 704 (52.3%) | 196 (43.4%) | 103 (54.2%) | 27 (41.5%) | 31 (55.4%) |
| Health Professional | 45 (3.3%) | 25 (5.5%) | 6 (3.2%) | 1 (1.5%) | 3 (5.4%) |
| Pharmacist | 22 (1.6%) | 17 (3.8%) | 3 (1.6%) | 3 (4.6%) | 2 (3.6%) |
| Physician | 470 (34.9%) | 169 (37.4%) | 57 (30.0%) | 29 (44.6%) | 20 (35.7%) |
| Unknown | 105 (7.8%) | 45 (10.0%) | 21 (11.1%) | 5 (7.7%) | 0 (0%) |
| Reported Countries, n (%) |  |  |  |  |  |
| US | 1269 (94.3%) | 405 (89.6%) | 176 (92.6%) | 64 (98.5%) | 51 (91.1%) |
| Non-US | 77(5.7%) | 47(10.4%) | 14(7.4%) | 1(1.5%) | 5(8.9%) |
| Serious Cases, n (%) |  |  |  |  |  |
| Yes | 468 (34.8%) | 240 (53.1%) | 50 (26.3%) | 12 (18.5%) | 55 (98.2%) |
| No | 878 (65.2%) | 212 (46.9%) | 140 (73.7%) | 53 (81.5%) | 1 (1.8%) |
| Reporting Year, n (%) |  |  |  |  |  |
| 2015 | 5 (0.4%) | 2 (0.4%) | 4 (2.1%) | 2 (3.1%) | 0 (0%) |
| 2016 | 91 (6.8%) | 50 (11.1%) | 27 (14.2%) | 11 (16.9%) | 8 (14.3%) |
| 2017 | 171 (12.7%) | 80 (17.7%) | 16 (8.4%) | 3 (4.6%) | 1 (1.8%) |
| 2018 | 511 (38.0%) | 118 (26.1%) | 72 (37.9%) | 25 (38.5%) | 11 (19.6%) |
| 2019 | 108 (8.0%) | 59 (13.1%) | 13 (6.8%) | 7 (10.8%) | 2 (3.6%) |
| 2020 | 78 (5.8%) | 37 (8.2%) | 8 (4.2%) | 7 (10.8%) | 4 (7.1%) |
| 2021 | 60 (4.5%) | 24 (5.3%) | 5 (2.6%) | 1 (1.5%) | 9 (16.1%) |
| 2022 | 58 (4.3%) | 17 (3.8%) | 10 (5.3%) | 1 (1.5%) | 4 (7.1%) |
| 2023 | 90 (6.7%) | 25 (5.5%) | 14 (7.4%) | 4 (6.2%) | 5 (8.9%) |
| 2024 | 122 (9.1%) | 24 (5.3%) | 12 (6.3%) | 3 (4.6%) | 8 (14.3%) |
| 2025 | 52 (3.9%) | 16 (3.5%) | 9 (4.7%) | 1 (1.5%) | 4 (7.1%) |

**S4 Baseline Characteristics of the Chinese Coronary Heart Disease Patients**

| Parameters | PCSK9 Inhibitors（n=601） | Non-PCSK9 Inhibitors（n=602） | *P* |
| --- | --- | --- | --- |
| Age, year | 59.96±9.73 | 59.86±9.97 | 0.868 |
| BMI, kg/m² | 26.62±13.29 | 26.42±11.19 | 0.775 |
| Gender (n, %) |  |  | 0.232 |
| Male | 459(76.37) | 477(79.24) |  |
| Female | 142(23.63) | 125(20.76) |  |
| DBP (mmHg) | 77.43±12.51 | 76.70±11.73 | 0.297 |
| SBP (mmHg) | 129.95±18.65 | 128.29±18.92 | 0.124 |
| Heart Rate (cpm) | 78.53±12.04 | 78.66±11.60 | 0.848 |
| Hypertension (n, %) |  |  | 0.932 |
| Yes | 316 (52.58) | 318 (52.82) |  |
| No | 285 (47.42) | 284 (47.18) |  |
| Smoking (n, %) |  |  | 0.088 |
| Yes | 382 (46.92) | 253 (42.03) |  |
| No | 319 (53.08) | 349 (57.97) |  |
| Drinking (n, %) |  |  | 0.487 |
| Yes | 204 (33.94) | 193 (32.06) |  |
| No | 397 (66.06) | 409 (67.94) |  |
| Leukocyte (×10^9/L) | 7.48±2.11 | 7.16±2.01 | 0.007 |
| Neutrophil (×10^9/L) | 4.83±1.97 | 4.42±1.79 | <0.001 |
| Lymphocyte (×10^9/L) | 1.92±0.65 | 1.96±065 | 0.253 |
| Monocyte (×10^9/L) | 0.55±0.21 | 0.54±0.18 | 0.801 |
| Platelet (×10^9/L) | 233.52±58.79 | 234.22±66.75 | 0.848 |
| Hemoglobin (g/L) | 141.11±15.50 | 141.60±16.34 | 0.594 |
| ALT (U/L) | 31.86±19.05 | 31.39±25.74 | 0.719 |
| UA (μmol/L) | 332.80 (271.82, 384.15) | 330.65 (277.71, 388.05) | 0.250 |
| TG (mmol/L) | 2.00±1.45 | 1.91±1.66 | 0.357 |
| TC (mmol/L) | 4.10±1.19 | 3.87±1.12 | <0.001 |
| HDL-C (mmol/L) | 0.95±0.22 | 0.93±0.24 | 0.356 |
| LDL-C (mmol/L) | 2.61±0.93 | 2.37±0.86 | <0.001 |
| CK-MB (IU/L) | 0.79(0.49, 1.23) | 0.75(0.52, 1.10) | 0.571 |
| Mb (ug/L) | 30.20 (22.80, 39.87) | 29.59 (23.37, 38.50) | 0.684 |
| ALP (U/L) | 78.36±18.89 | 77.76±34.00 | 0.703 |
| APTT(S) | 32.18±6.27 | 32.38±4.93 | 0.557 |
| INR | 1.00±0.09 | 1.00±0.08 | 0.796 |
| D-Dimer (ng/ml) | 92.00 (58.00, 132.50) | 90.00 (58.75, 136.00) | 0.338 |
| FDP (ug/ml) | 0.82 (0.55, 1.12) | 0.78 (0.49, 1.07) | 0.070 |
| PTA(%) | 103.79±18.71 | 103.24±19.32 | 0.613 |
| Follow-up (month) | 17.5 (11.63, 22.92) | 16.73 (10.92, 24.65) | 0.972 |

BMI body mass index, DBP diastolic blood pressure, SBP systolic blood pressure, ALT alanine aminotransferase, UA uric acid,

TG triglyceride, TC total cholesterol, HDL-C high-density lipoprotein cholestetol, LDL low-density lipoprotein cholestetol,

CKMB Creatine Kinase-MB, Mb Myoglobin, ALP Alkaline Phosphatase, APTT Activated Partial Thromboplastin Time,

INR International Normalized Ratio, FDP Fibrinogen Degradation Products, PTA  Prothrombin Time Activity
